# Supplementary material for: Tailored Surgical Stabilization of Rib Fractures Matters More Than the Number of Fractured Ribs
Source: J Pers Med. 2022 Nov 4;12(11):1844. doi: 10.3390/jpm12111844 (PMC9698685; doi:10.3390/jpm12111844)
Supplement: Supplementary file 1 [file jpm-12-01844-s001.zip › Table S1.pdf]

Preoperative variables between unmatched groups

	SSRF (-) N = 256	SSRF (+) N = 177	*P*
**Age, y**	58.0 (40.0-70.0)	56.0 (45.5-64.5)	0.52
**Male/Female**	157/99	106/71	0.76
**Transferal**	79 (30.9)	60 (33.9)	0.51
**Charlson Comorbidity Index**	2.0 (0.0-4.0)	2.0 (0.0-3.0)	0.67
**Trauma mechanism**			0.02
**Vehicle-to-vehicle collision**	118 (46.1)	112 (63.3)	
**Vehicle-to-pedestrian collision**	15 (5.9)	6 (3.4)	
**Single-vehicle collision**	66 (25.8)	32 (18.1)	
**Fall**	52 (20.3)	26 (14.7)	
**Crush**	3 (1.2)	1 (0.6)	
**Assault**	2 (0.8)	0 (0.0)	
**Glasgow Coma Scale**	15.0 (15.0-15.0)	15.0 (15.0-15.0)	0.05
**Injury Severity Score**	20.5 (13.0-29.0)	17.0 (13.0-24.0)	0.07
**Head/Neck**			
**Median (IQR)**	2.0 (0.0-3.0)	0.0 (0.0-2.0)	0.02
**AIS ≥3**	83 (32.4)	30 (16.9)	<0.01
**Face**			
**Median (IQR)**	0.0 (0.0-1.0)	0.0 (0.0-0.0)	<0.01
**AIS ≥3**	5 (2.0)	1 (0.6)	0.41
**Thorax**			
**Median (IQR)**	3.0 (3.0-3.0)	3.0 (3.0-3.0)	<0.01
**AIS ≥3**	203 (79.3)	177 (100.0)	<0.01
**Abdomen**			
**Median (IQR)**	0.0 (0.0-2.0)	0.0 (0.0-2.0)	0.05

**AIS ≥3**	58 (22.7)	26 (14.7)	0.04
**Extremity**			
**Median (IQR)**	2.0 (0.0-2.0)	2.0 (0.0-2.0)	0.68
**AIS ≥3**	56 (21.9)	24 (13.6)	0.03
**External**			
**Median (IQR)**	0.0 (0.0-0.0)	0.0 (0.0-0.0)	0.19
**AIS ≥3**	1 (0.4)	2 (1.1)	0.57
**Number of ribs broken**	4.0 (2.0-6.0)	7.0 (5.0-9.0)	<0.01
**Fractured side**			0.52
**Unilateral**	214 (83.6)	152 (85.9)	
**Bilateral**	42 (16.4)	25 (14.1)	
**Presence of a flail segment radiologically**	16 (6.3)	72 (40.7)	<0.01
**Requiring mechanical ventilation**	10 (3.9)	26 (14.7)	<0.01
**Associated intrathoracic injury**			
**Lung contusion/laceration**	103 (40.2)	68 (38.4)	0.70
**Pneumothorax**	88 (34.4)	94 (53.1)	<0.01
**Hemothorax**	112 (43.8)	151 (85.3)	<0.01
**Cardiac injury**	5 (2.0)	2 (1.1)	0.71
**Great vessels injury**	9 (3.5)	1 (0.6)	0.05
**Soft tissue injury**	21 (8.2)	43 (24.3)	<0.01
**Concurrent sternal fracture**	8 (3.1)	8 (4.5)	0.45
**Concurrent ipsilateral clavicular fracture**	65 (25.4)	83 (46.9)	<0.01
**Concurrent ipsilateral scapular fracture**	27 (10.5)	37 (20.9)	<0.01

Flail chest, the presence of three or more contiguous ribs fractured in two or more places; AIS, abbreviated injury scale; SSRF, surgical stabilization of rib fractures
