# Supplementary material for: Tailored Surgical Stabilization of Rib Fractures Matters More Than the Number of Fractured Ribs
Source: J Pers Med. 2022 Nov 4;12(11):1844. doi: 10.3390/jpm12111844 (PMC9698685; doi:10.3390/jpm12111844)
Supplement: Supplementary file 1 [file jpm-12-01844-s001.zip › Table S2.pdf]

Outcomes between unmatched groups

	SSRF (-)<br>N = 256	SSRF (+)<br>N = 177	*P*
**Ventilator days, d**	6.0 (2.0-12.0)<br>N = 82	5.0 (2.0-9.0)<br>N = 70	0.34
**Length of ICU stay, d**	6.0 (3.0-14.0)<br>N = 119	6.0 (3.0-12.0)<br>N = 93	0.82
**Length of hospital stay, d**	9.0 (5.3-18.8)	13.0 (9.0-19.5)	<0.01
**Time from trauma to ambulation, d**	5.0 (3.0-11.0)	3.0 (1.0-7.5)	<0.01
**Nonprocedure-related pulmonary complication count**	48 (18.8)	13 (7.3)	<0.01
**Tracheostomy**	10 (3.9)	0 (0.0)	<0.01
**Mortality**	5 (2.0)	0 (0.0)	0.08

SSRF, surgical stabilization of rib fractures

1
