# Supplementary material for: Tailored Surgical Stabilization of Rib Fractures Matters More Than the Number of Fractured Ribs
Source: J Pers Med. 2022 Nov 4;12(11):1844. doi: 10.3390/jpm12111844 (PMC9698685; doi:10.3390/jpm12111844)
Supplement: Supplementary file 1 [file jpm-12-01844-s001.zip › Table S3.pdf]

A comparison of the perioperative variables of SSRF group with and without more than six fractured ribs

	Fractured ribs ≤6<br>N = 85	Fractured ribs >6<br>N = 92	p
**Age at time of surgery, y**	57.0 (48.0-64.0)	55.5 (44.0-65.0)	0.79
**Male/Female**	50/35	56/36	0.78
**Transferal**	30 (35.3)	30 (32.6)	0.71
**Charlson Comorbidity Index**	2 (0-3)	2 (0-3)	0.78
**Medication required hypertension**	22 (25.9)	29 (31.5)	
**Coronary artery disease**	7 (8.2)	6 (6.5)	
**Atrial fibrillation**	3 (3.5)	2 (2.2)	
**Peripheral artery disease**	2 (2.4)	0 (0.0)	
**Heart failure (ACC/AHA ≥ stage C)**	1 (1.2)	0 (0.0)	
**Medication required diabetes mellitus**	23 (27.1)	14 (15.2)	
**Chronic kidney disease (eGFR < 60 mL/min)**	2 (2.4)	3 (3.3)	
**Liver cirrhosis**	2 (2.4)	1 (1.1)	
**Connective tissue disease**	0 (0.0)	3 (3.3)	
**Chronic obstructive pulmonary disease/Asthma**	1 (1.2)	1 (1.1)	
**Psychiatric disorders**	3 (3.5)	4 (4.3)	
**Malignancy**	5 (5.9)	6 (6.5)	
**Trauma mechanism**			0.91
**Vehicle-to-vehicle collision**	54 (63.5)	58 (63.0)	
**Vehicle-to-pedestrian collision**	3 (3.5)	3 (3.3)	
**Single-vehicle collision**	16 (18.8)	16 (17.4)	
**Fall**	12 (14.1)	14 (15.2)	
**Crush**	0 (0.0)	1 (1.1)	
**Glasgow Coma Scale**	15 (15-15)	15 (15-15)	0.19
**Injury Severity Score**	17.0 (13.0-22.0)	21.5 (17.0-26.5)	<0.01
**Head/Neck**			
**Median (IQR)**	0 (0-2)	0 (0-2)	0.70

**AIS ≥3**	14 (16.5)	16 (17.4)	0.87
**Face**			
**Median (IQR)**	0 (0-0)	0 (0-0)	0.44
**AIS ≥3**	0 (0.0)	1 (1.1)	>0.99
**Thorax**			
**Median (IQR)**	3 (3-3)	3 (3-4)	<0.01
**AIS ≥3**	85 (100.0)	92 (100.0)	NA
**Abdomen**			
**Median (IQR)**	0 (0-2)	0 (0-2)	0.03
**AIS ≥3**	8 (9.4)	18 (19.6)	0.06
**Extremity**			
**Median (IQR)**	2 (0-2)	2 (2-2)	<0.01
**AIS ≥3**	4 (4.7)	20 (21.7)	<0.01
**External**			
**Median (IQR)**	0 (0-0)	0 (0-0)	0.59
**AIS ≥3**	0 (0.0)	2 (2.2)	0.50
**Number of ribs broken**	5 (4-6)	8 (7-10)	<0.01
**Fractured side**			<0.01
**Right**	33 (38.8)	36 (39.1)	
**Left**	47 (55.3)	36 (39.1)	
**Both**	5 (5.9)	20 (21.7)	
**Position on the involved ribs (502 vs. 1000)**			0.12
**Anterior**	7 (1.4)	16 (1.6)	
**Anterolateral**	112 (22.3)	234 (23.4)	
**Posterolateral**	347 (69.1)	643 (64.3)	
**Posterior**	36 (7.2)	107 (10.7)	
**Presence of a flail segment radiologically**	15 (17.6)	57 (62.0)	<0.01
**Requiring mechanical ventilation**	2 (2.4)	24 (26.1)	<0.01
**Associated intrathoracic injury**			

**Lung contusion/laceration**	24 (28.2)	44 (47.8)	<0.01
**Pneumothorax**	37 (43.5)	57 (62.0)	0.01
**Hemothorax**	68 (80.0)	83 (90.2)	0.06
**Cardiac injury**	1 (1.2)	1 (1.1)	>0.99
**Great vessels**	0 (0.0)	1 (1.1)	>0.99
**Soft tissue**	16 (18.8)	27 (29.3)	0.10
**Concurrent sternal fracture**	4 (4.7)	4 (4.3)	>0.99
**Concurrent ipsilateral clavicular fracture**	36 (42.4)	47 (51.1)	0.25
**Concurrent ipsilateral scapular fracture**	13 (15.3)	24 (26.1)	0.08
**Time from trauma to rib fixation, d**	4.0 (2.0-6.0)	4.5 (3.0-7.0)	0.05
**Surgical indication**			
**Flail chest**	15 (17.8)	57 (62.0)	<0.01
**nonflail fractures with respiratory compromise**	8 (9.4)	6 (6.5)	0.48
**Chest wall deformity/severely displaced fractures**	5 (5.9)	6 (6.5)	0.86
**Thoracotomy for associated thoracic injury (e.g. hemothorax, lung laceration)**	53 (62.4)	67 (72.8)	0.14
**Intractable pain despite appropriate analgesia**	62 (72.9)	57 (62.0)	0.12
**Number of surgically fixated rib fractures**	4 (3-4)	5 (4-6)	<0.01
**Fracture fixation ratio**	0.8 (0.6-0.9)	0.6 (0.4-0.7)	<0.01
**Mechanical ventilation during anesthesia (n = 169)**			0.96
**Single lung ventilation**	62 (72.9)	61 (72.6)	
**Double lung ventilation**	23 (27.1)	23 (27.4)	
**Approach side**			0.09
**Right**	36 (42.4)	50 (54.3)	
**Left**	49 (57.6)	40 (43.5)	
**Both**	0 (0.0)	2 (2.2)	
**Incision**			0.75
**Thoracotomy**	24 (28.2)	24 (26.1)	
**Video-assisted mini-thoracotomy**	61 (71.8)	68 (73.9)	

**Combined operation**	44 (51.8)	46 (50.0)	0.82
**Operation time, min**	131 (111-162)	187 (140-222)	<0.01
**Blood loss, ml**	0 (0-150)	100 (0-300)	<0.01

Flail chest, the presence of three or more contiguous ribs fractured in two or more places; fracture fixation ratio, total fixated ribs divided by total fractured ribs; SSRF, surgical stabilization of rib fractures
