# Supplementary material for: Tailored Surgical Stabilization of Rib Fractures Matters More Than the Number of Fractured Ribs
Source: J Pers Med. 2022 Nov 4;12(11):1844. doi: 10.3390/jpm12111844 (PMC9698685; doi:10.3390/jpm12111844)
Supplement: Supplementary file 1 [file jpm-12-01844-s001.zip › Table S4.pdf]

P value of univariate analyses of SSRF (+) group

	Logistic regression for NPRCs	Linear regression for log-ventilator days	Linear regression for log-ICU stay	Linear regression for log-hospital stay
**Age at time of surgery, y**	0.03	0.65	0.87	0.60
**Sex (female vs. male)**	0.20	0.54	0.35	0.65
**Transferal**	0.34	0.79	0.62	0.44
**Charlson comorbidity index**	0.14	0.33	0.80	0.98
**Non-car accidents vs. Car accidents**	0.42	0.15	0.87	0.45
**Glasgow coma scale**	<0.01	<0.01	<0.01	<0.01
**Injury severity score**	0.08	0.01	<0.01	<0.01
**Number of ribs broken**	0.35	0.20	0.04	<0.01
**Fractured side (bilateral vs. unilateral)**	>0.99	0.49	0.04	0.04
**Presence of a flail segment radiologically**	0.12	0.29	0.33	<0.01
**Requiring mechanical ventilation**	<0.01	<0.01	<0.01	<0.01
**Associated intrathoracic injury**				
**Lung contusion/laceration**	>0.99	0.45	<0.01	<0.01
**Pneumothorax**	0.28	0.37	0.14	0.22
**Hemothorax**	0.47	0.86	0.91	0.75
**Cardiac injury**	>0.99	0.62	0.87	0.96
**Great vessels injury**	>0.99	0.53	0.39	0.14
**Soft tissue injury**	0.57	0.62	0.56	0.05
**Concurrent sternal fracture**	>0.99	0.75	0.86	0.46
**Concurrent ipsilateral clavicular fracture**	0.24	0.28	0.72	0.74
**Concurrent ipsilateral scapular fracture**	0.61	0.27	0.29	0.42
**Time from trauma to rib fixation, d**	0.34	<0.01	<0.01	<0.01
**Number of surgically fixated rib fractures**	0.04	0.09	0.17	<0.01
**Fracture fixation ratio**	0.27	0.89	0.47	0.27
**Double lumen vs. Single lumen intubation**	0.17	0.13	0.06	0.02

**Approach side (bilateral vs. unilateral)**	>0.99	0.45	0.29	0.12
**Video-assisted mini-thoracotomy vs. Thoracotomy**	0.34	0.04	0.04	0.13
**Concomitant surgery for other injuries**	0.36	0.20	0.75	0.55
**Operation time, min**	0.02	0.05	0.20	<0.01
**Blood loss during SSRF**	0.02	0.01	0.01	<0.01

NPRCs, Non-Procedural Postoperative Pulmonary Complications; SSRF, surgical stabilization of rib fractures
