# Supplementary material for: Tailored Surgical Stabilization of Rib Fractures Matters More Than the Number of Fractured Ribs
Source: J Pers Med. 2022 Nov 4;12(11):1844. doi: 10.3390/jpm12111844 (PMC9698685; doi:10.3390/jpm12111844)
Supplement: Supplementary file 1 [file jpm-12-01844-s001.zip › Table S5.pdf]

P value of univariate analyses of SSRF (-) group

	Logistic regression for NPRCs	Linear regression for log-ventilator days	Linear regression for log-ICU stay	Linear regression for log-hospital stay
**Age, y**	0.21	0.08	0.67	0.44
**Sex (female vs. male)**	0.74	0.64	0.94	0.31
**Transferal**	0.70	0.44	0.69	<0.01
**Charlson comorbidity index**	0.07	0.01	0.17	0.59
**Non-car accidents vs. Car accidents**	0.58	0.12	0.98	0.89
**Glasgow coma scale**	<0.01	0.02	<0.01	<0.01
**Injury severity score**	<0.01	0.13	0.02	<0.01
**Number of ribs broken**	0.62	0.09	0.02	0.02
**Fractured side (bilateral vs. unilateral)**	0.85	0.30	0.05	0.02
**Presence of a flail segment radiologically**	0.75	0.98	0.73	0.43
**Requiring mechanical ventilation**	<0.01	<0.01	<0.01	<0.01
**Associated intrathoracic injury**				
**Lung contusion/laceration**	0.41	0.76	0.37	0.30
**Pneumothorax**	0.19	0.08	0.04	0.13
**Hemothorax**	0.09	0.36	0.59	0.84
**Cardiac injury**	0.77	0.23	0.64	0.53
**Great vessels injury**	0.02	0.46	0.16	<0.01
**Soft tissue injury**	0.26	0.45	0.39	0.19
**Concurrent sternal fracture**	0.44	0.95	0.33	0.95
**Concurrent ipsilateral clavicular fracture**	0.97	0.71	0.92	0.96
**Concurrent ipsilateral scapular fracture**	0.62	0.42	0.62	0.63

NPRCs, Non-Procedural Postoperative Pulmonary Complications; SSRF, surgical stabilization of rib fractures
